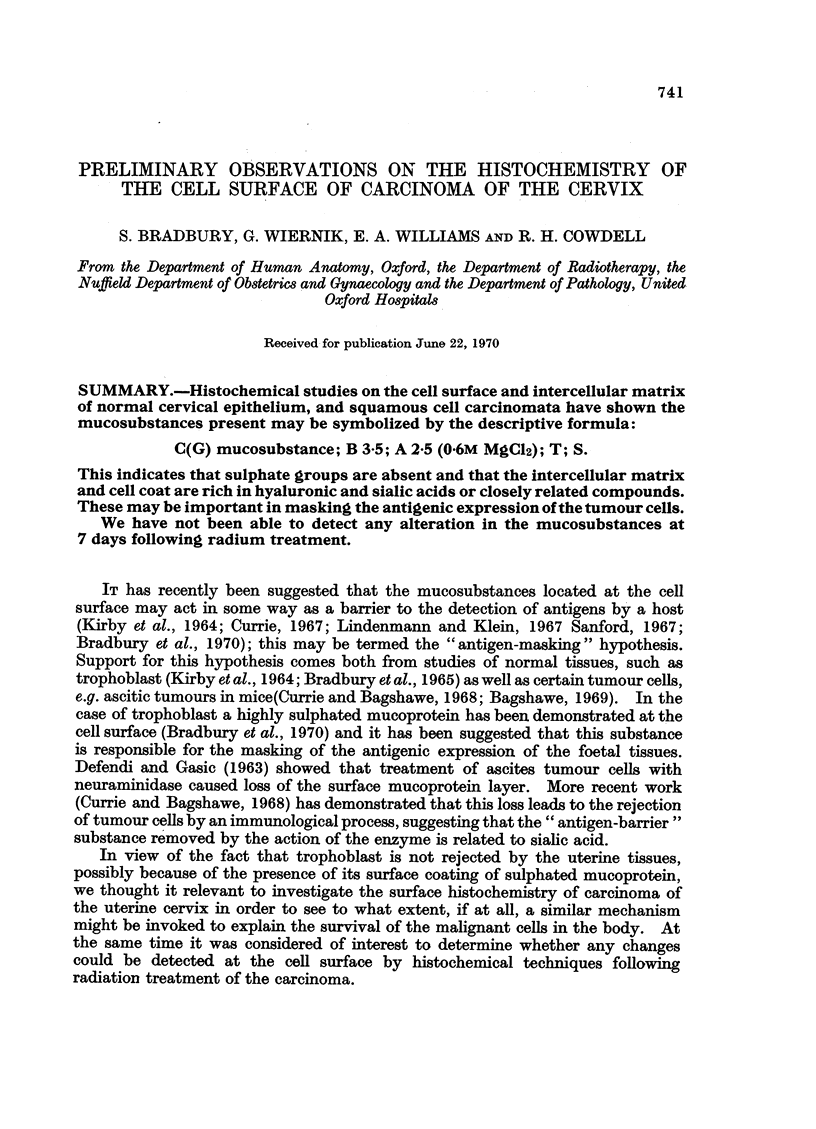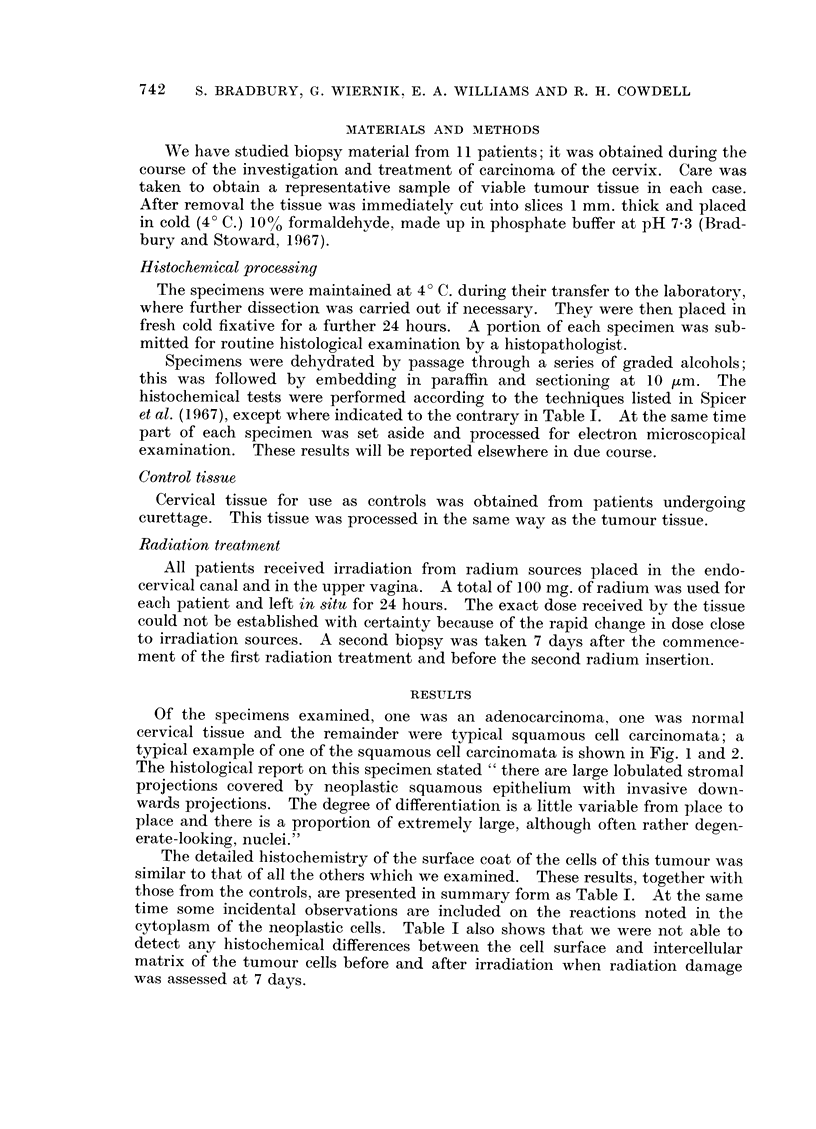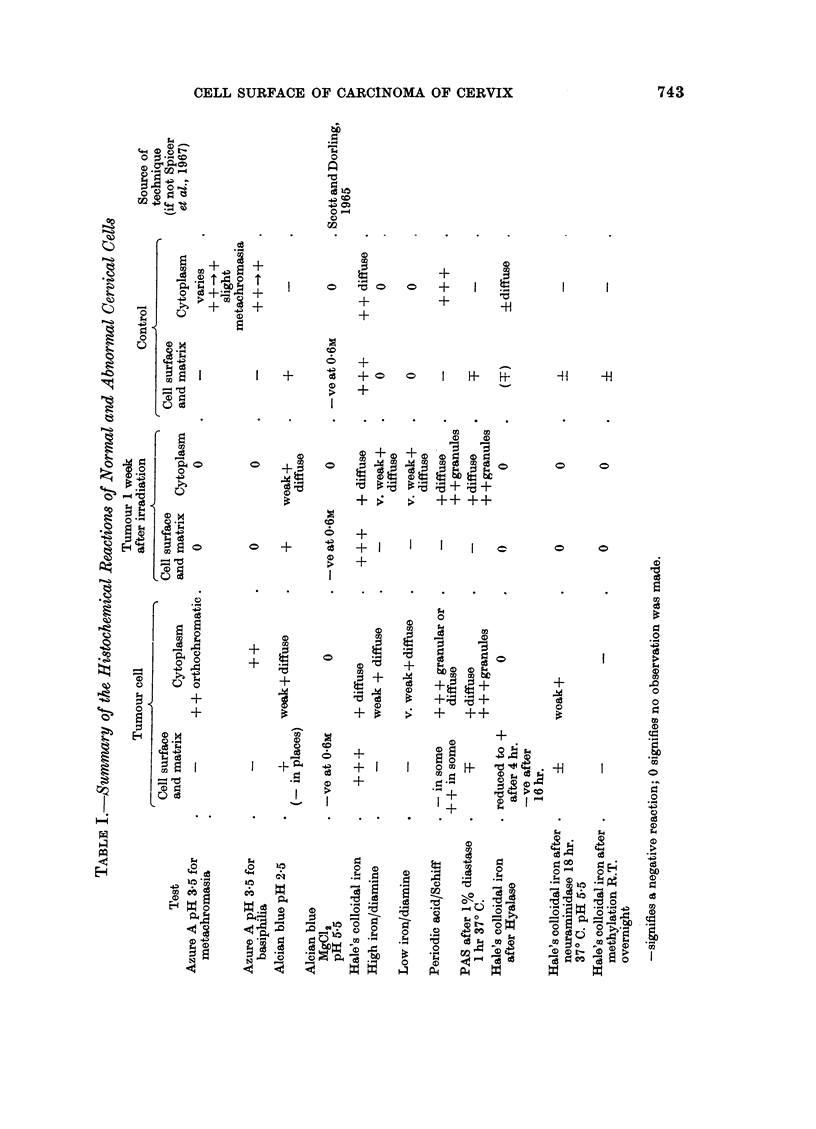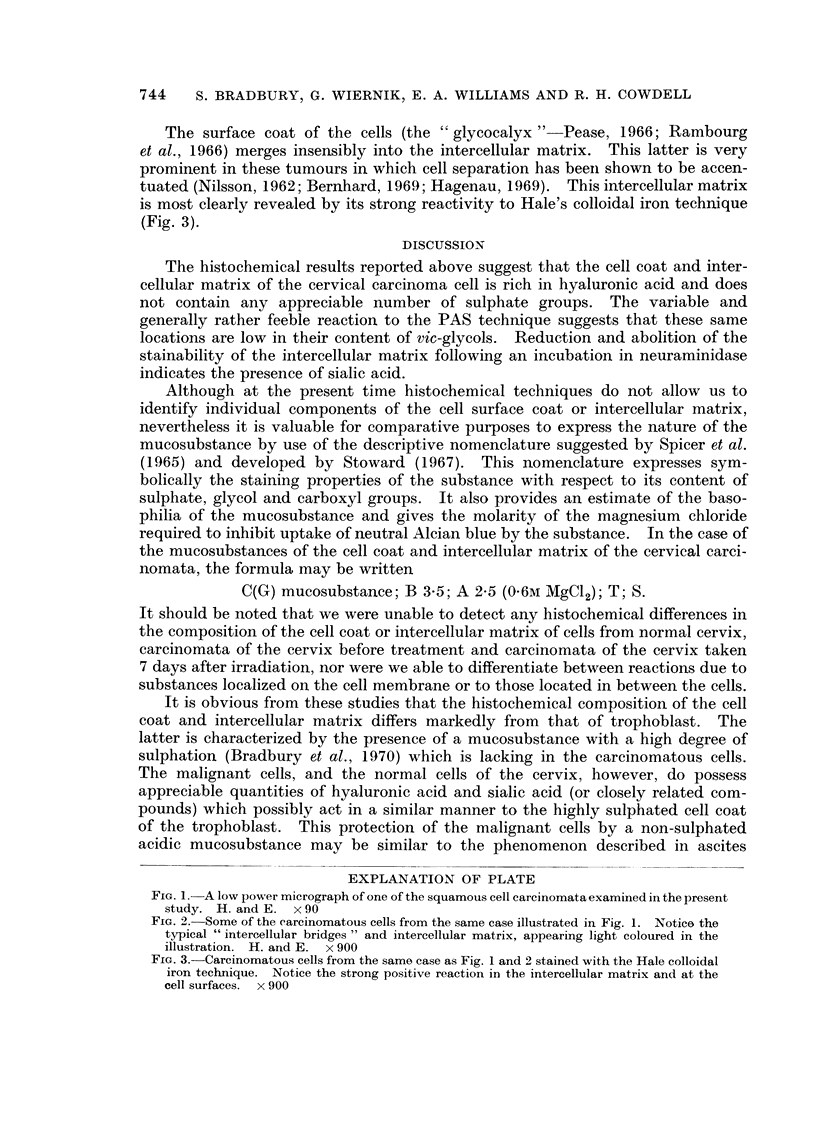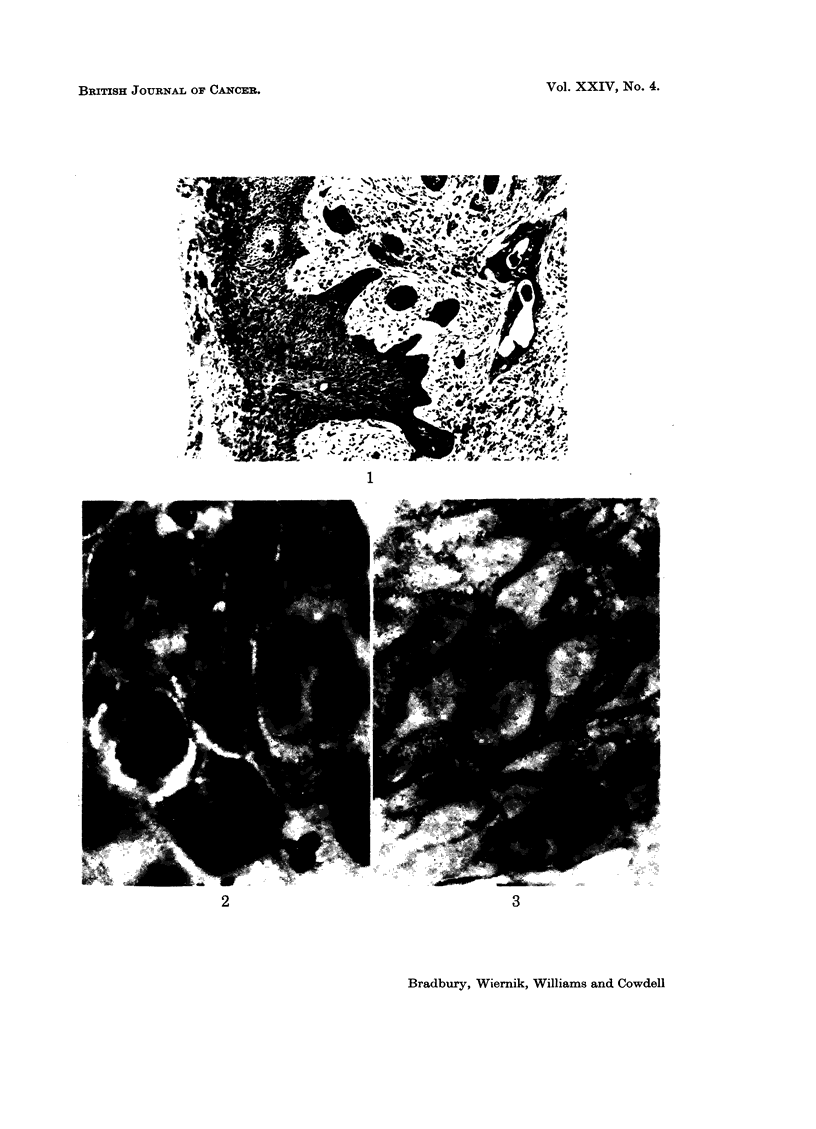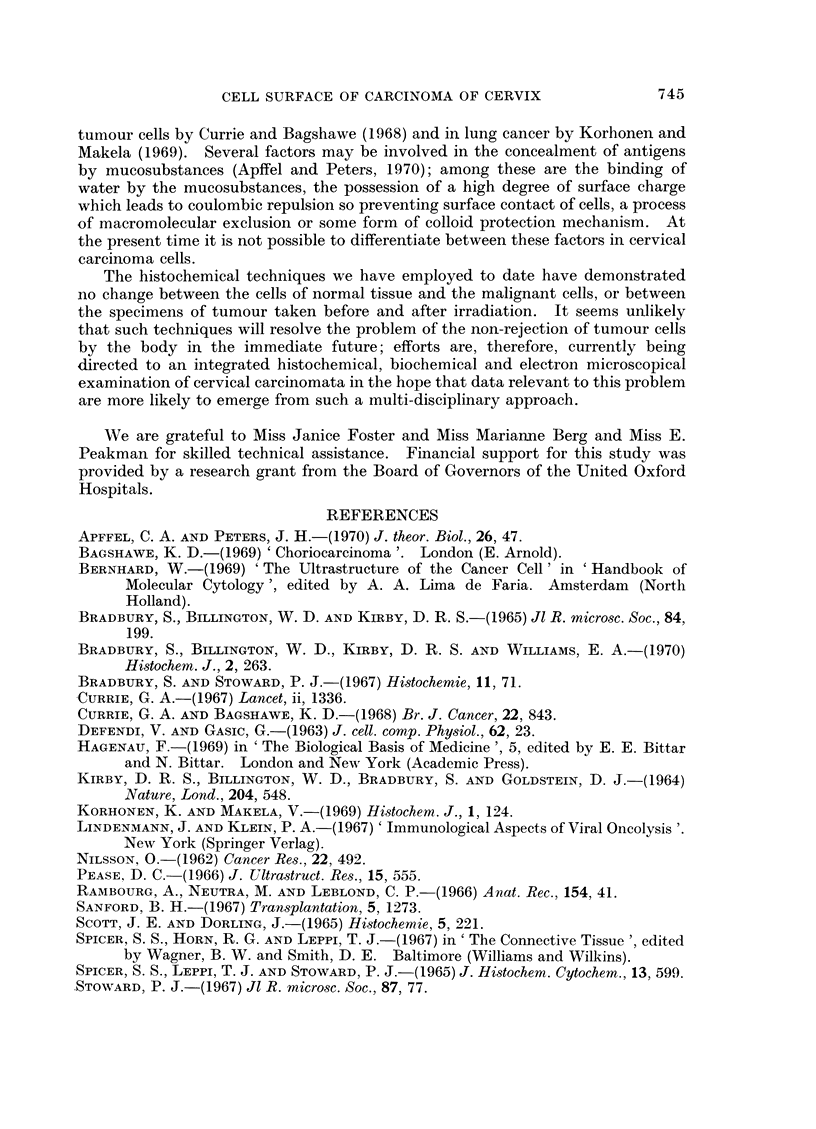# Preliminary Observations on the Histochemistry of the Cell Surface of Carcinoma of the Cervix

**DOI:** 10.1038/bjc.1970.88

**Published:** 1970-12

**Authors:** S. Bradbury, G. Wiernik, E. A. Williams, R. H. Cowdell

## Abstract

**Images:**


### PRELIMINARY OBSERVATIONS ON THE HISTOCHEMISTRY OF THE CELL SURFACE OF CARCINOMA OF THE CERVIX

S. BRADBURY, G. WIERNIK, E. A. WILLIAMS AND R. H. COWDELL

*From the Department of Human Anatomy, Oxford, the Department of Radiotherapy, the Nuffield Department of Obstetrics and Gynaecology and the Department of Pathology, United Oxford Hospitals*

Received for publication June 22, 1970

**SUMMARY.—Histochemical studies on the cell surface and intercellular matrix of normal cervical epithelium, and squamous cell carcinomata have shown the mucosubstances present may be symbolized by the descriptive formula:**

**C(G) mucosubstance; B 3·5; A 2·5 (0·6M $MgCl_2$); T; S.**

**This indicates that sulphate groups are absent and that the intercellular matrix and cell coat are rich in hyaluronic and sialic acids or closely related compounds. These may be important in masking the antigenic expression of the tumour cells.**

**We have not been able to detect any alteration in the mucosubstances at 7 days following radium treatment.**

IT has recently been suggested that the mucosubstances located at the cell surface may act in some way as a barrier to the detection of antigens by a host (Kirby *et al.*, 1964; Currie, 1967; Lindenmann and Klein, 1967 Sanford, 1967; Bradbury *et al.*, 1970); this may be termed the "antigen-masking" hypothesis. Support for this hypothesis comes both from studies of normal tissues, such as trophoblast (Kirby *et al.*, 1964; Bradbury *et al.*, 1965) as well as certain tumour cells, *e.g.* ascitic tumours in mice(Currie and Bagshawe, 1968; Bagshawe, 1969). In the case of trophoblast a highly sulphated mucoprotein has been demonstrated at the cell surface (Bradbury *et al.*, 1970) and it has been suggested that this substance is responsible for the masking of the antigenic expression of the foetal tissues. Defendi and Gasic (1963) showed that treatment of ascites tumour cells with neuraminidase caused loss of the surface mucoprotein layer. More recent work (Currie and Bagshawe, 1968) has demonstrated that this loss leads to the rejection of tumour cells by an immunological process, suggesting that the " antigen-barrier " substance removed by the action of the enzyme is related to sialic acid.

In view of the fact that trophoblast is not rejected by the uterine tissues, possibly because of the presence of its surface coating of sulphated mucoprotein, we thought it relevant to investigate the surface histochemistry of carcinoma of the uterine cervix in order to see to what extent, if at all, a similar mechanism might be invoked to explain the survival of the malignant cells in the body. At the same time it was considered of interest to determine whether any changes could be detected at the cell surface by histochemical techniques following radiation treatment of the carcinoma.

### MATERIALS AND METHODS

We have studied biopsy material from 11 patients; it was obtained during the course of the investigation and treatment of carcinoma of the cervix. Care was taken to obtain a representative sample of viable tumour tissue in each case. After removal the tissue was immediately cut into slices 1 mm. thick and placed in cold (4° C.) 10% formaldehyde, made up in phosphate buffer at pH 7·3 (Bradbury and Stoward, 1967).

### *Histochemical processing*

The specimens were maintained at 4° C. during their transfer to the laboratory, where further dissection was carried out if necessary. They were then placed in fresh cold fixative for a further 24 hours. A portion of each specimen was submitted for routine histological examination by a histopathologist.

Specimens were dehydrated by passage through a series of graded alcohols; this was followed by embedding in paraffin and sectioning at 10 $\mu$m. The histochemical tests were performed according to the techniques listed in Spicer *et al.* (1967), except where indicated to the contrary in Table I. At the same time part of each specimen was set aside and processed for electron microscopical examination. These results will be reported elsewhere in due course.

### *Control tissue*

Cervical tissue for use as controls was obtained from patients undergoing curettage. This tissue was processed in the same way as the tumour tissue.

### *Radiation treatment*

All patients received irradiation from radium sources placed in the endocervical canal and in the upper vagina. A total of 100 mg. of radium was used for each patient and left *in situ* for 24 hours. The exact dose received by the tissue could not be established with certainty because of the rapid change in dose close to irradiation sources. A second biopsy was taken 7 days after the commencement of the first radiation treatment and before the second radium insertion.

### RESULTS

Of the specimens examined, one was an adenocarcinoma, one was normal cervical tissue and the remainder were typical squamous cell carcinomata; a typical example of one of the squamous cell carcinomata is shown in Fig. 1 and 2. The histological report on this specimen stated " there are large lobulated stromal projections covered by neoplastic squamous epithelium with invasive downwards projections. The degree of differentiation is a little variable from place to place and there is a proportion of extremely large, although often rather degenerate-looking, nuclei."

The detailed histochemistry of the surface coat of the cells of this tumour was similar to that of all the others which we examined. These results, together with those from the controls, are presented in summary form as Table I. At the same time some incidental observations are included on the reactions noted in the cytoplasm of the neoplastic cells. Table I also shows that we were not able to detect any histochemical differences between the cell surface and intercellular matrix of the tumour cells before and after irradiation when radiation damage was assessed at 7 days.

TABLE I.—*Summary of the Histochemical Reactions of Normal and Abnormal Cervical Cells*

| Test | Tumour cell | | Tumour 1 week after irradiation | | Control | | Source of technique (if not Spicer *et al.*, 1967) |
|---|---|---|---|---|---|---|---|
| | Cell surface and matrix | Cytoplasm | Cell surface and matrix | Cytoplasm | Cell surface and matrix | Cytoplasm | |
| Azure A pH 3·5 for metachromasia | — | ++ orthochromatic | 0 | 0 | — | varies ++→+ slight metachromasia | |
| Azure A pH 3·5 for basiphilia | — | ++ | 0 | 0 | — | ++→+ | |
| Alcian blue pH 2·5 | + (— in places) | weak+diffuse | + | weak+ diffuse | + | — | |
| Alcian blue $MgCl_2$ pH 5·5 | —ve at 0·6M | 0 | —ve at 0·6M | 0 | —ve at 0·6M | 0 | Scott and Dorling, 1965 |
| Hale's colloidal iron | +++ | + diffuse | +++ | + diffuse | +++ | ++ diffuse | |
| High iron/diamine | — | weak + diffuse | — | v. weak+ diffuse | 0 | 0 | |
| Low iron/diamine | — | v. weak+diffuse | — | v. weak+ diffuse | 0 | 0 | |
| Periodic acid/Schiff | — in some ++ in some | +++ granular or diffuse | — | +diffuse ++granules | — | +++ | |
| PAS after 1% diastase 1 hr 37° C. | ∓ | +diffuse +++granules | — | +diffuse ++granules | ∓ | — | |
| Hale's colloidal iron after Hyalase | reduced to + after 4 hr. —ve after 16 hr. | 0 | 0 | 0 | (∓) | ±diffuse | |
| Hale's colloidal iron after neuraminidase 18 hr. 37° C. pH 5·5 | ± | weak+ | 0 | 0 | ± | — | |
| Hale's colloidal iron after methylation R.T. overnight | — | — | 0 | 0 | ± | — | |

—signifies a negative reaction; 0 signifies no observation was made.

The surface coat of the cells (the " glycocalyx "—Pease, 1966; Rambourg *et al.*, 1966) merges insensibly into the intercellular matrix. This latter is very prominent in these tumours in which cell separation has been shown to be accentuated (Nilsson, 1962; Bernhard, 1969; Hagenau, 1969). This intercellular matrix is most clearly revealed by its strong reactivity to Hale's colloidal iron technique (Fig. 3).

### DISCUSSION

The histochemical results reported above suggest that the cell coat and intercellular matrix of the cervical carcinoma cell is rich in hyaluronic acid and does not contain any appreciable number of sulphate groups. The variable and generally rather feeble reaction to the PAS technique suggests that these same locations are low in their content of *vic*-glycols. Reduction and abolition of the stainability of the intercellular matrix following an incubation in neuraminidase indicates the presence of sialic acid.

Although at the present time histochemical techniques do not allow us to identify individual components of the cell surface coat or intercellular matrix, nevertheless it is valuable for comparative purposes to express the nature of the mucosubstance by use of the descriptive nomenclature suggested by Spicer *et al.* (1965) and developed by Stoward (1967). This nomenclature expresses symbolically the staining properties of the substance with respect to its content of sulphate, glycol and carboxyl groups. It also provides an estimate of the basophilia of the mucosubstance and gives the molarity of the magnesium chloride required to inhibit uptake of neutral Alcian blue by the substance. In the case of the mucosubstances of the cell coat and intercellular matrix of the cervical carcinomata, the formula may be written

$$\text{C(G) mucosubstance; B 3·5; A 2·5 (0·6}\textsc{m}\ MgCl_2\text{); T; S.}$$

It should be noted that we were unable to detect any histochemical differences in the composition of the cell coat or intercellular matrix of cells from normal cervix, carcinomata of the cervix before treatment and carcinomata of the cervix taken 7 days after irradiation, nor were we able to differentiate between reactions due to substances localized on the cell membrane or to those located in between the cells.

It is obvious from these studies that the histochemical composition of the cell coat and intercellular matrix differs markedly from that of trophoblast. The latter is characterized by the presence of a mucosubstance with a high degree of sulphation (Bradbury *et al.*, 1970) which is lacking in the carcinomatous cells. The malignant cells, and the normal cells of the cervix, however, do possess appreciable quantities of hyaluronic acid and sialic acid (or closely related compounds) which possibly act in a similar manner to the highly sulphated cell coat of the trophoblast. This protection of the malignant cells by a non-sulphated acidic mucosubstance may be similar to the phenomenon described in ascites

### EXPLANATION OF PLATE

FIG. 1.—A low power micrograph of one of the squamous cell carcinomata examined in the present study. H. and E. ×90

FIG. 2.—Some of the carcinomatous cells from the same case illustrated in Fig. 1. Notice the typical " intercellular bridges " and intercellular matrix, appearing light coloured in the illustration. H. and E. ×900

FIG. 3.—Carcinomatous cells from the same case as Fig. 1 and 2 stained with the Hale colloidal iron technique. Notice the strong positive reaction in the intercellular matrix and at the cell surfaces. ×900

1

2

3

tumour cells by Currie and Bagshawe (1968) and in lung cancer by Korhonen and Makela (1969). Several factors may be involved in the concealment of antigens by mucosubstances (Apffel and Peters, 1970); among these are the binding of water by the mucosubstances, the possession of a high degree of surface charge which leads to coulombic repulsion so preventing surface contact of cells, a process of macromolecular exclusion or some form of colloid protection mechanism. At the present time it is not possible to differentiate between these factors in cervical carcinoma cells.

The histochemical techniques we have employed to date have demonstrated no change between the cells of normal tissue and the malignant cells, or between the specimens of tumour taken before and after irradiation. It seems unlikely that such techniques will resolve the problem of the non-rejection of tumour cells by the body in the immediate future; efforts are, therefore, currently being directed to an integrated histochemical, biochemical and electron microscopical examination of cervical carcinomata in the hope that data relevant to this problem are more likely to emerge from such a multi-disciplinary approach.

We are grateful to Miss Janice Foster and Miss Marianne Berg and Miss E. Peakman for skilled technical assistance. Financial support for this study was provided by a research grant from the Board of Governors of the United Oxford Hospitals.

### REFERENCES

APFFEL, C. A. AND PETERS, J. H.—(1970) *J. theor. Biol.*, **26**, 47.

BAGSHAWE, K. D.—(1969) ‘ Choriocarcinoma ’. London (E. Arnold).

BERNHARD, W.—(1969) ‘ The Ultrastructure of the Cancer Cell ’ in ‘ Handbook of Molecular Cytology ’, edited by A. A. Lima de Faria. Amsterdam (North Holland).

BRADBURY, S., BILLINGTON, W. D. AND KIRBY, D. R. S.—(1965) *Jl R. microsc. Soc.*, **84**, 199.

BRADBURY, S., BILLINGTON, W. D., KIRBY, D. R. S. AND WILLIAMS, E. A.—(1970) *Histochem. J.*, **2**, 263.

BRADBURY, S. AND STOWARD, P. J.—(1967) *Histochemie*, **11**, 71.

CURRIE, G. A.—(1967) *Lancet*, ii, 1336.

CURRIE, G. A. AND BAGSHAWE, K. D.—(1968) *Br. J. Cancer*, **22**, 843.

DEFENDI, V. AND GASIC, G.—(1963) *J. cell. comp. Physiol.*, **62**, 23.

HAGENAU, F.—(1969) in ‘ The Biological Basis of Medicine ’, 5, edited by E. E. Bittar and N. Bittar. London and New York (Academic Press).

KIRBY, D. R. S., BILLINGTON, W. D., BRADBURY, S. AND GOLDSTEIN, D. J.—(1964) *Nature, Lond.*, **204**, 548.

KORHONEN, K. AND MAKELA, V.—(1969) *Histochem. J.*, **1**, 124.

LINDENMANN, J. AND KLEIN, P. A.—(1967) ‘ Immunological Aspects of Viral Oncolysis ’. New York (Springer Verlag).

NILSSON, O.—(1962) *Cancer Res.*, **22**, 492.

PEASE, D. C.—(1966) *J. Ultrastruct. Res.*, **15**, 555.

RAMBOURG, A., NEUTRA, M. AND LEBLOND, C. P.—(1966) *Anat. Rec.*, **154**, 41.

SANFORD, B. H.—(1967) *Transplantation*, **5**, 1273.

SCOTT, J. E. AND DORLING, J.—(1965) *Histochemie*, **5**, 221.

SPICER, S. S., HORN, R. G. AND LEPPI, T. J.—(1967) in ‘ The Connective Tissue ’, edited by Wagner, B. W. and Smith, D. E. Baltimore (Williams and Wilkins).

SPICER, S. S., LEPPI, T. J. AND STOWARD, P. J.—(1965) *J. Histochem. Cytochem.*, **13**, 599.

STOWARD, P. J.—(1967) *Jl R. microsc. Soc.*, **87**, 77.